# IgG4-Related Sclerosing Disease Causing Spinal Cord Compression: The First Reported Case in Literature

**DOI:** 10.1155/2019/3618510

**Published:** 2019-06-18

**Authors:** Nooraldin Merza, Ahmed Taha, John Lung, Anthony W. Benderman, Stephen E. Wright

**Affiliations:** ^1^Department of Internal Medicine, Texas Tech University Health Sciences Center, Amarillo, TX, USA; ^2^School of Medicine, Texas Tech University Health Sciences Center, Amarillo, TX, USA; ^3^Department of Pathology, Veterans Affairs Medical Center, Amarillo, TX, USA; ^4^Department of Hematology and Oncology Medicine, Veterans Affairs Medical Center, Amarillo, TX, USA

## Abstract

Immunoglobulin G4-related disease (IgG4-RD) is known for forming soft tissue mass lesions that may have compressive effects. It is an extremely rare disease that most frequently affects the pancreas causing autoimmune pancreatitis. It can also affect the gallbladder, salivary glands, and lacrimal glands causing respective organ-specific complications. In our report, we describe an IgG4-RD case that affected the spinal cord. A 60-year-old female presented with cervical spinal cord compression caused by IgG4-RD leading to several neurological deficits. Pathological examination of the excisional biopsy of the mass revealed dense lymphoplasmacytic cells infiltration and stromal fibrosis with IgG4 and plasma cells. The patient showed a dramatic response to the administration of systemic steroids with almost resolution of her neurological symptoms. This case highlights the first case in literature for IgG4-RD of the extradural tissue causing spinal compression. Hereby, we also demonstrate the dramatic response of IgG4-RD to the administration of systemic steroids as the patient had no recurrence after 5 years of close follow-up, the longest reported period of follow-up reported in the literature to date.

## 1. Background

IgG4-related disease (IgG4-RD) is an extremely rare condition that can affect the pancreas, liver, salivary glands, kidneys, lungs, central nervous system (CNS), and the heart. It is diagnosed based on the clinical presentation, hematological data, and histopathological criteria. Very limited data is available about the CNS involvement in IgG4-RD because it is an extremely entity of the disease.

The manifestations of IgG4-RD when it affects the CNS depends typically on the location of the sclerotic mass. IgG4-RD have been previously described in the literature as it affected the pituitary gland and manifested as hypophysitis [1], the meninges and manifested as intracranial hypertrophic pachymeningitis [2–4], or the brain parenchyma and presented as an inflammatory pseudotumor [5]. We, hereby, present the first case in literature to describe IgG4-RD of the spinal extradural tissue. We also demonstrate the dramatic response of the disease to the administration of systemic steroids as the patient had no recurrence after 5 years of close follow-up.

## 2. Case Presentation

A 60-year-old Caucasian female with medical history of obstructive sleep apnea, seasonal allergies, and osteoarthritis who presented with weakness and numbness in all four extremities for 4 weeks. Initially, she had bilateral burning pain at the tips of her fingers and toes that progressed later to pin-and-needle paresthesia. The paresthesia was associated with low grade fever, bowel and bladder incontinence, and vague dull neck pain. Her home medications are acetaminophen, methocarbamol, and vitamin D supplementation and she is allergic to aspirin, calcium, cortisone meperidine, phenytoin, gabapentin, ibuprofen, naproxen, penicillin, salicylate, and sulfa drugs. She denied alcohol, tobacco, and illicit drug use.

She has a past surgical history of 2 caesarian sections, hemorrhoidectomy 31 years ago, and splenectomy after a remote vehicle accident 35 years ago. 5 months prior to this presentation, she underwent decompressive laminectomy for a C1-C5 cervical mass originating from the dorsal part of the cervical epidural space. The pathology report from the resected mass revealed an inflammatory mass with extensive collagenized background and a polymorphous-appearing cell infiltrates with a mixture of small lymphoid cells, plasmacytoid cells, very occasional eosinophils, and neutrophils.

At admission, she was alert and oriented and maintaining normal vital signs. Physical examination revealed that her neck was supple without lymphadenopathy but was significant for neck tenderness. CNS examination revealed intact cranial nerves, 3/5 power strength and 2+ reflexes in upper extremities bilaterally, 2/5 strength and 3+ reflexes in lower extremities bilaterally, intact sensation in all four extremities, and no saddle anesthesia. The rest of the physical examination was unremarkable.

**Figure 1:**
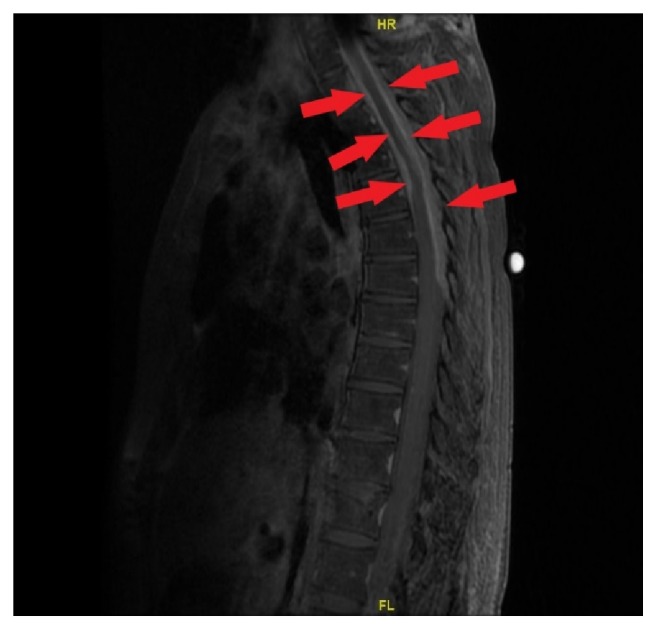
T1-weighted Thoracic spine Magnetic Resonance Imaging showing diffuse extradural mass effect and enhancement from the T1 level to the T5 level anteriorly and to the T7 level posteriorly; most prominent at the T4-T5 level where there is marked spinal canal stenosis and mass effect upon the spinal cord.

CBC, serum electrolytes, and blood chemistry were unremarkable. Magnetic resonance imaging (MRI) of the thoracic spine showed T2-diffuse enhancement of extradural soft tissue mass with marked spinal canal stenosis, most prominent at the level of T4-T5 (Figure 1). Whole body positron emission tomography (PET) scan showed no evidence of malignancy with no abnormality demonstrated in the spleen, liver, pancreas, salivary or adrenal glands. There were prominent bilateral jugular nodes with a standard uptake value of 2.6 on the right and 2.3 on the left. Lumbar puncture was performed and cerebrospinal fluid (CSF) cytological analysis revealed mature lymphocytes and monocytes with no malignant cells. Flow cytometry showed no immunophenotypic evidence of non-Hodgkin's lymphoma.

**Figure 2:**
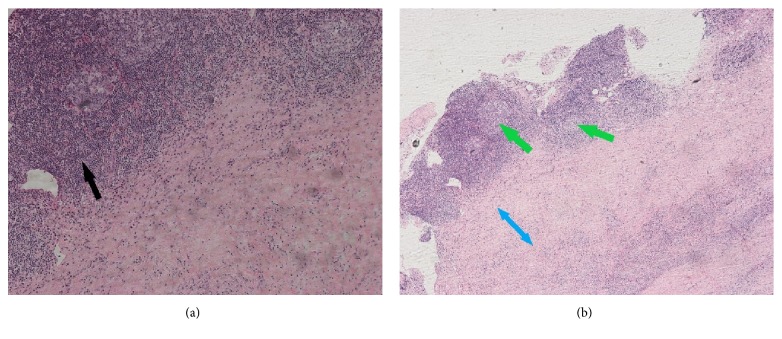
Hematoxylin and Eosin (H&E) section of the epidural mass (200x magnification power for (a) and 100x magnification power for (b)) showing (a) chronic inflammatory infiltrates, predominantly mononuclear infiltrate, lymphoid hyperplasia (black arrow) and plasma cells emulating the classic storiform fibrosis pattern; no phlebitis obliterans were identified. (b) Dense collagen deposition and dense fibrous tissue formation (blue arrow) with focal germinal center formation (green arrows).

A laminectomy of T2 through T6 with excisional biopsy of the epidural mass was performed. The histological examination of the extradural mass chronic inflammatory infiltration composed of lymphocytes, plasma cells, histiocytes, neutrophils, eosinophils, mast cells (Figure 2(a)). It also showed dense collagen deposition and fibrous tissue formation with focal germinal center formation (Figure 2(b)). The classic storiform fibrosis pattern was identified in the biopsy material, but no phlebitis obliterans was identified. KAPPA-ISH and lambda-ISH stains showed poly-clonality with an equal mixture of kappa positive and lambda positive plasma cells. AFB and PAS stains show no mycobacterial or fungal organisms. Immunohistochemistry was performed to evaluate the nature of the plasma cells, and it revealed an IgG4+/IgG+ ratio of 47% which was diagnostic for IgG4-RD. Serum immunoglobulins were then checked and showed a total IgG of 17.65 g/L and elevated IgG4 fragment (2.07 g/L).

## 3. Treatment/Follow Up

After the surgery, the patient was discharged to an acute rehabilitation facility and then home but she continued to have quadriparesis, bowel and bladder incontinence, and limited ambulation due to motor weakness and poor sensation. It was almost 6 weeks until the final pathology report was received that confirmed the diagnosis of IgG4-RD, after which she was started on weekly pulse doses of oral dexamethasone 40 mg split into 4 doses. Due to her corticosteroid allergy, she was pretreated with 50 mg diphenhydramine 30 minutes before the administration of dexamethasone.

**Figure 3:**
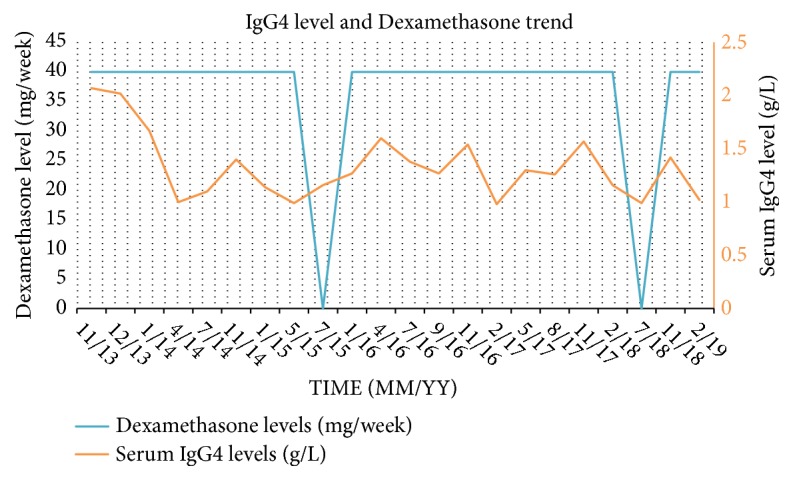
Double y-axis chart showing the IgG4 trends (orange line) and the Dexamethasone compliance (blue line) over time. Please note the immediate rise in serum IgG4 levels following the fall in Dexamethasone dosage.

A dramatic clinical improvement was achieved, particularly in her quadriparesis, after the administration of systemic corticosteroids. She was able to walk with walker within 3-4 months, then independently in about a year. Her hand Fine motor skills have improved tremendously and she is currently able to perform knitting. Her bowel and bladder dysfunction also recovered completely. Over the course of 5 years of close follow up, she was kept on dexamethasone 40 mg weekly preceded with diphenhydramine and she did not experience any disease relapse. Annual follow-up MRI images have shown no disease recurrence and IgG levels remained suppressed for 5 years after the initial diagnosis and treatment, the longest follow-up period for IgG4-RD reported. Serum IgG4 levels in relation to her compliance to Dexamethasone is outlined in Figure 3.

## 4. Discussion

IgG4-RD is a rare disease which was first recognized as one of the causes of autoimmune and sclerosing cholangitis [6]. Subsequently, extra-pancreatic organ involvement was reported, these include the liver (e.g., sclerosing cholangitis when involving intrahepatic ducts), salivary glands (e.g., IgG4-sialadenitis and its variants), kidneys (e.g., interstitial nephritis), lungs, and rarely the CNS or cardiac tissue [1]. The diagnosis is so challenging, and in most cases, it ends up reported as an idiopathic inflammatory disorder. IgG4-RD can also affect several organ systems making the differential diagnosis list too lengthy.

The diagnosis of IgG4-RD relies on clinical, hematologic, and histopathologic criteria. These include patients' presentation with characteristic diffuse or localized masses in single or multiple organs, elevated serum IgG4 levels >1.35 g/L, marked fibrosis with lymphocytic and plasmacytic infiltration with positive immunohistochemical staining for IgG4 on histological examination. IgG4+/IgG ratio >40% and >50 IgG4+ plasma cells/high power field are also very suggestive for IgG4-RD on pathological examination [7].

The immunological mechanism of IgG4-related sclerosing disease is not completely understood. The prevailing theory is the one that involves molecular mimicry based on an immune response to self-antigens that results in an increased regulatory T-cell and type 2 T-helper (TH2) cell response that results in persistent inflammation [8]. IgG4 antibodies bind to the epithelial lining of various organs and react with autoantigens causing the chronic inflammatory reaction required to develop IgG4-RD [6]. However, the role of IgG4 in the pathogenesis is unclear, though higher levels of IgG4 clearly correlate with disease severity [9].

IgG4-RD has been reported to affect the CNS in literature. The most common CNS manifestation of IgG4-RD is hypertrophic pachymeningitis where it lead to dural hypertrophy (and thickening) which will eventually lead to symptoms such as headaches, cranial nerve dysfunctions, sensory and/or motor symptoms depending to the location, either intracranially or spinally [3]. Meningeal involvement of IgG4-RD might be confused with many differential diagnoses including; Idiopathic hypertrophic pachymeningitis, inflammatory myofibroblastic tumor, lymphoma, granulomatosis with polyangiitis, giant-cell arteritis, Langerhans-cell histiocytosis, and sarcoidosis [10].

17 cases were reported in the literature where IgG4-RD hypertrophic pachymeningitis (Table 1) was causing nerve compression symptoms lasting for months [11–13]. Our case report is the first case in literature where IgG4-RD caused an extradural mass complicated by spinal cord compression.

Unlike other systemic inflammatory diseases such as sarcoidosis or systemic scleroderma, the immunological manifestations of IgG4-RD are reversible with corticosteroids. Once the diagnosis has been made, the disease often dramatically responds to systemic corticosteroids. Some patients may not meet the histopathological criteria for IgG sclerosing disease, yet still, show clinical improvement with glucocorticoids [14]. A dramatic clinical improvement was achieved in our patient, particularly in her quadriparesis, after the administration of systemic corticosteroids. She was able to walk independently in just a few months. Her bowel and bladder dysfunction also recovered completely, but they took little longer, i.e. 6-9 months.

Currently, there is no consensus about the dose or duration of systemic corticosteroid therapy for IgG4-RD. Some physicians treat with systemic steroids temporarily for several months, while some patients in Japan get corticosteroids permanently [15]. We elected to keep our patient on life-long steroids due to the severe symptoms at initial presentation, the significant disease morbidity, and the immediate elevation of IgG4 levels at the time that Dexamethasone was briefly held (Figure 3) [16, 17]. The life-long administration of corticosteroids in a pulsatile manner i.e. Dexamethasone 40 mg/week in our patient has been shown similar outcomes, but with more acceptable side-effect profile [18]. Intermittent glucocorticoid administration at a high dose has a strong effect due to 100% saturation of cytosolic receptors; however, the effect would last only for a short period because receptor occupation rapidly reverts to the original value unless a new dose is given [18].

Therefore, a single high dose is unlikely to have sustained effect. Overall, the effects of corticosteroid pulses appear to include downregulation of activation of immune cells and proinflammatory cytokine production, leading to reduced expression of adhesion molecules and reduced movement of neutrophils into sites of inflammation. These effects are qualitatively similar to those seen with anti-TNF-alpha therapy [19]. Corticosteroids are currently recommended orally for IgG4-RD according to the international consensus guidance [20]. Other routes of glucocorticoids administration, such as intrathecal and epidural routes of administration have risks of arachnoiditis, spinal cord injury, and spinal infection [17]. More recently, experiments in mice indicate that glucocorticoids may have a similar role in B lymphocytes [16, 21–24]; nonetheless, physicians are currently challenged with the choice of which glucocorticoids to use, what dose, and for how long to use them.

## 5. Conclusions

Though extremely rare, spinal cord compression could be the initial presentation of IgG4-RD. The IgG4-RD disease process can be rapidly ameliorated with systemic corticosteroid use, even in the late stages of the disease. Early diagnosis of this rare IgG4-RD disease entity, extradural spinal cord compression, and early treatment with systemic corticosteroids can decrease the disease mortality and morbidity and improve patients' quality of life [25].

## Figures and Tables

**Table 1:** 

Case	Patient Age	Gender	Clinical presentation	Presence of extra-spinal disease	Treatment	Outcome
Chan et al. 2009	37	Male	Bilateral lower extremity weakness, numbness, unsteady gait × 2 weeks	Not reported	Not reported	Not reported

Choi et al. 2010	46	Female	Progressive bilateral lower extremity weakness and numbness × 2 weeks	Not reported	Steroids	Improved exam to ‘near normal' after initial surgery; progression 2 months post op required second resection (subtotal) with full recovery; no recurrence at 8 months

Lindstrom et al. 2010	55	Male	Cord compression, C3-C7 mass	Not reported	Steroids; Radiation Therapy	“Doing well” at 15 month follow up

Lindstrom et al. 2010	63	Male	Bilateral hand numbness, C2-C3 mass	Not reported	Not reported	Lost to follow-up

Tajima et al. 2012	64	Male	Gradually progressive and worsening dysphagia for 1 month	Slight swelling in both kidneys-renal	Steroids	Improvement of symptoms and MRI findings at 3 weeks

Wallace et al. 2013	32	Male	Weakness of dorsum of right foot	Not reported	Not reported	Lost to follow-up

Ezzeldin et al. 2014	55	Male	Diplegia of lower extremities and T4/T5 sensory level for 2 weeks	Not reported	Steroids	Able to ambulate at discharge (timing not specified)

Kim et al. 2014	52	Female	Sudden weakness of bilateral lower extremities for 2 days; paraplegia, urinary retention	None	Steroids	Unchanged after 2 months

Gu et al. 2016	43	Male	Neck pain for 15 days, bilateral lower extremity numbness/weakness, bowel/bladder dysfunction for 4 days	Patient declined testing	Not reported	Resolution of symptoms at 6 months post-op

Radotra et al. 2016	50	Male	Progressive bilateral lower extremity weakness for 6 months	Not reported	Steroids	Marginal improvement in strength 7 months post-op

Radotra et al. 2016	19	Male	back and left lower extremity pain for 1 year; subtle knee extensor weakness bilaterally	Not reported	Steroids	Pain free at 6 months; stable exam

Ferreira et al. 2016	57	Female	Worsening low back pain for 2.5 years; bilateral lower extremity numbness and weakness	None	Steroids	Recurrence and progressive paresis later improved after third resection and long-term steroids

Lu et al. 2016	55	Male	Diffuse numbness and weakness for 6 months; constipation and dysuria for 7 days	Not reported	Steroids, cyclophosphamide	Recovered defecation/urination at 20 days; ambulated independently at 5 months

Rumalla et al. 2017	50	Male	Rapidly progressive severe back pain at T6 level for 3 months; acute onset paraplegia, T6 sensory level	Right lung associated with adjacent vertebral involvement	Steroids	Near normal lower extremity strength 2 months post-op

Williams et al. 2017	46	Female	Worsening neck pain and bilateral upper extremity paresthesias and weakness for 4 months	Not reported	Steroids, azathioprine	Improving strength at 6 month post-op visit

Bridges et al. 2017	68	Male	Intermittent thoracic spine pain for 3 years, progressive trunk numbness and bilateral lower extremity numbness for 6 months, dysequilibrium	None	Steroids	Walking independently with resolution of pain at 3 months post-op

Winkel et al. 2018	48	Female	Lower back pain, neurogenic claudication, right lower extremity radiculopathy, extradural mass L2-L3 level	None	Steroids	Asymptomatic at 1 year follow-up
